# A qualitative reflexive thematic analysis of innovation and regulation in hearing health care

**DOI:** 10.1186/s12916-024-03627-1

**Published:** 2024-09-27

**Authors:** Isabelle Boisvert, Samantha Cruz Rivera, Jennifer Smith-Merry, Barbara Molony-Oates, Emily Kecman, Sarah E. Hughes

**Affiliations:** 1 Sydney School of Health Sciences, Faculty of Medicine and Health, The University of Sydney, Sydney, Australia; 2 Centre for Disability Research and Policy, Faculty of Medicine and Health, The University of Sydney, Sydney, Australia; 3 Centre for Patient Reported Outcome Research, Department of Applied Health Sciences, University of Birmingham, Birmingham, UK; 4 Birmingham Health Partners Centre for Regulatory Science, Birmingham, UK; 5 Health Research Authority (HRA), London, UK

**Keywords:** Hearing loss, Deafness, Innovation, Digital health, Regulation, Qualitative research, Thematic analysis, Stakeholder perspectives, Healthcare, Disability policy

## Abstract

**Background:**

The hearing health sector is an example of a health sector that is experiencing a period of rapid innovation driven by digital technologies. These innovations will impact the types of interventions and services available to support the communication of deaf and hard-of-hearing individuals. This study explored the perceptions of informed participants on the topic of innovation and regulation within hearing healthcare in Australia and the United Kingdom (UK).

**Methods:**

Participants (*N* = 29, Australia [*n* = 16], UK [*n* = 13]) were purposively sampled and joined one of two online workshops. Participants included adults with hearing loss and family members, hearing health professionals, academics/researchers, representatives of hearing device manufacturers, regulators and policymakers. Workshop data were analysed using reflexive thematic analysis.

**Results:**

Participants conceptualised the hearing health sector as a network of organisations and individuals with different roles, knowledge and interests, in a state of flux driven by innovation and regulation. Innovation and regulation were perceived as mechanisms to ensure quality and mitigate risk within a holistic approach to care. Innovations encompassed technological as well as non-technological innovations of potential benefit to consumers. Participants agreed it was essential for innovation and regulation to be congruent with societal values. Critical to ethical congruence was the involvement of consumers throughout both innovation and regulation stages, and the use of innovation and regulation to tackle stigma and reduce health disparities. Participants expressed the desire for accessible and inclusive innovation in the context of fair, transparent and trustworthy commercial practices.

**Conclusions:**

This study explored how stakeholders within the hearing health sector understand and make sense of innovation and the role of regulation. Overall, and despite reservations relating to health care professionals’ changing roles and responsibilities, innovation and regulation were conceptualised as beneficial when situated in the context of holistic, whole-person, models of care. The results of this study will inform considerations to support the development and implementation of innovations and regulation within the hearing sector and across other health sectors influenced by technological advances.

**Supplementary Information:**

The online version contains supplementary material available at 10.1186/s12916-024-03627-1.

## Background

Digital innovation is transforming health care with opportunities to increase care accuracy, efficiency and accessibility [1, 2]. Digital innovation in health care can include the use of apps and sensors on wearable devices to collect patient data (within authentic, simulated, remote, augmented or virtual environments); data integration with information collected from other digital networks; comparison of individuals’ data with large datasets; and the use of artificial intelligence to support diagnoses and prognoses as well as the recommendation and monitoring of treatment options [1]. Digital innovations can promote self-management and direct-to-consumer care, and therefore hold the potential to lessen healthcare costs [3, 4]. While more research is needed in this area, digital innovations are often stated as part of the solution to address shortage and high workload of health professionals [2]. The increased digitisation occurring across different fields of health care is concomitant to a range of other health innovations, including advances in biotechnologies (e.g. genetics, pharmacology, stem cell treatments) and innovative health service approaches (e.g. remote, self-managed, direct-to-consumer or person-centred approaches). This article focuses particularly on hearing health care—a field which has been transformed by digital technologies and other innovations over 30 years [5–8]. The article examines this context to illustrate how innovations can remodel health care services and systems, and the implications of these innovations from the perspective of stakeholders within the field.

With innovation comes not only opportunities but also risks, and, broadly, it is the role of health regulation to limit potential risks (e.g. physical, psychological or financial risks) which might arise from the provision of a health service or product. Designing valuable regulations, however, is complex and multi-layered and may be perceived as impeding the innovative process [9, 10]. Balancing access to innovation while limiting potential risk therefore often requires compromise [9, 11]. In the case of digital health innovation and regulation, managing risks also involves the management of digital data and the safe transfer of information between different types of technologies or sectors (for example, heart rate data capture shared via Bluetooth or Wi-Fi networks). Regulators must consider the opportunities and risks not only in relation to specific innovations and specific health contexts, but also how different innovations and regulatory systems interact beyond the boundaries of a particular field [12].

Digital innovation began in the 1950s with the digitisation of telecommunication and computing technologies, the main technologies used within hearing devices. Digital hearing aids were therefore one of the earliest wearable and digitised health technologies. For the last 30 years, digital innovations have led the ongoing transformation of hearing care by providing improved means for hearing screening and monitoring, as well as tools for hearing loss prevention, diagnosis and intervention [5, 7]. Hearing devices such as hearing aids and cochlear implants can now connect to a range of Bluetooth-enabled or smart technologies, sharing similarities with popular lifestyle products such as advanced noise-cancelling earphones that stream from smartphones. Hearing devices rely on microphones that capture sounds in the environment but can now also be fitted with biosensors such as those found on smartwatches. The field of hearing care is also a fertile field for advances in biotechnologies and service delivery models. For example, the implanted electrode array of cochlear implants can now be used to deliver drugs within the cochlea to limit potential hair cell damage [13], and speech processors of cochlear implants can be programmed remotely, without the need to attend a clinic in person. Processors can now also be controlled through a patients’ smartphone and transfer information to their clinical files [14]. The extent to which the benefits of cochlear implantation are realised, however, remains dependent on how effectively services are provided at different levels: from the surgeons implanting the internal component, to audiologists programming the devices, to speech-language pathologists or hearing therapists supporting aural and communication rehabilitation and device use. This includes learning how to use the device and monitoring whether adjustment, repairs or basic maintenance (e.g. battery changes) are required. Practitioners must constantly update their services as new knowledge, research and technology become available. Hearing care is therefore a useful context to examine how the interplay between a range of evolving innovations and regulations influences health services.

Beyond risks of harm and/or opportunities for patients, innovation can also be thought of through the lens of risks and opportunities for product and service developers and providers. In the context of hearing health care, one or more regulatory organisation may focus on the person *offering* or *receiving* the service (e.g. professional associations or health boards), other organisations may focus on the safety and efficacy of different product categories (e.g. general consumer versus medical devices), and others on regulating the way products and services are advertised or funded. Regulations developed by different groups responsible for different components of health services and product provision can therefore be misaligned due to the different interests at stake. The involvement of consumers during the development of products and services is also increasingly encouraged as it has been shown to improve health outcomes [15, 16]. Avoiding a siloed or disjointed approach to regulation and innovation therefore requires in-depth understanding and careful balancing of the perspectives of consumers, product developers and service providers, as well as policymakers. With these complexities in mind, this article focuses on the field of hearing health care, a field already accustomed to wearable digital health devices, and explores stakeholders’ perspectives on ways in which innovation and regulation are reshaping health services. This article further aims to derive practical recommendations from these integrated perspectives, to support the development of future innovation and regulation.

## Methods

### Participants

We sampled purposively for age, gender, degree of hearing loss, device use, whether sign language was used and, where relevant, years of professional experience, within and across multiple relevant parties in the hearing health sector in the United Kingdom (UK) and Australia. We recruited adults with hearing loss, family members, audiologists (from private and public sectors), regulators, policymakers, industry representatives, representatives of non-governmental charitable organisations and academics/researchers working in the fields of audiology and regulatory science. Study recruitment took place via promotion within social media channels (i.e. Twitter, LinkedIn). Snowball sampling was also used, with emails sent to known contacts of the researchers or to the generic email addresses of relevant organisations to identify relevant and interested participants. After having been provided with written information about the purpose and context of this study, participants provided informed consent via the web application Smart Survey and reconfirmed their consent verbally at the beginning of each workshop. Participants with hearing loss and family members received a gift voucher for their time and involvement.

### Data collection

Using Zoom videoconferencing software, two focus groups of a duration of 2 h each were held (one in Australia and one in the UK) in October and November 2022 respectively. In each country, the workshop was facilitated by a member of the research team local to the setting (Australia: IB, UK: SEH). A second member of the research team moderated the session and provided technical support with other research team members as observers/note-takers. Live speech-to-text reporters were available for both workshops. Auslan interpreting was provided for the Australian workshop. British Sign Language (BSL) was not requested by any UK participants.

Workshop discussions were recorded using the University of Birmingham’s Zoom recording function and verbatim transcripts were generated by the speech-to-text reporters. Pre-reading material, an agenda, a semi-structured topic guide and companion Padlet boards were developed for use in both workshops to ensure consistency. Participants received an information pack by email before the workshop. This information pack included the pre-readings, agenda and topic guide (see Supplementary file 1). The topics for the workshops were structured in two parts. The first part comprised some initial broad questions relating to participants’ views of current and emerging trends, changes within hearing health care and the role that innovation and regulation might play going forward. The second part of the topic guide presented participants with three vignettes or specific ‘scenarios’—these scenarios profiled different types of potential hearing care ‘clients’ with distinct characteristics, preferences and circumstances. The scenarios were accompanied by questions designed to stimulate discussion amongst the different stakeholders. Both the use of scenarios as a tool for generating rich discussions as well as the actual scenarios presented to participants in this study were modelled on the work presented in the Ida Institute’s Future Journey Report—in which a number of scenarios based on expected future health care trends within healthcare and hearing care were developed [17]. Participants could choose to contribute to the workshop using their voice, sign language or written text. The use of Padlet boards provided participants with a de-identified way to share their thoughts with the group. Participants could also communicate independently with the workshop moderator if they wished, who would then add to the Padlet board on their behalf. Finally, all participants were also offered the opportunity to discuss with the lead researchers before and after the workshops.

### Data analysis

All data sources (observer notes, verbatim transcripts, anonymised participant responses on Padlet boards) were downloaded and stored securely on the University of Birmingham server. Data that were not provided anonymously to the researchers were de-identified, and reflexive thematic analysis undertaken with all the data using a constructivist approach [18]. Reflexive thematic analysis is an updated description of Braun and Clarke’s thematic analyses approach [19, 20] and explicitly recognises the researchers’ subjectivity and reflexivity as they engage with and try to make sense of the data [20]. A constructivist approach acknowledges that the data gathered, and the interpretation of that data, are influenced by contextual factors [21]. Therefore, different perspectives can shape the knowledge that is constructed during the analysis. Annotation and analyses were performed using a combination of NVivo qualitative data analysis software and Microsoft Word. Initial familiarisation with the data was followed by first level, line-by-line coding. To enhance analytic rigour and to support comparison across settings, two researchers (IB and SEH) independently analysed a subset of the data to develop a set of initial codes. Codes were compared and harmonised via consensus discussion to develop a coding framework. Iterative cycles of coding and refining produced additional inductive codes that were added to the framework. Visual mapping techniques were used to explore relationships between codes and identify coding clusters to develop an initial set of themes. Initial themes were further refined by writing a synopsis for each theme. Consensus-building discussions enabled co-development of the final themes. An early iteration of the analysis, presented as a conference poster, was shared with some of the participants to confirm whether the findings represented the discussions of the workshop [22]. Participants were not provided access to the full transcripts of the interviews.

### Trustworthiness and reflexivity

This study was reported in accordance with the COnsolidated criteria for REporting Qualitative research (COREQ; see Supplementary File 2 [23]). Throughout the study, the research team engaged in reflexive discussions to acknowledge subjectivity and the researcher lens (IB is an academic and clinical audiologist interested in the information that supports hearing health intervention options; SEH is a researcher and speech-language pathologist with a special interest in hearing loss; SCR is a researcher with expertise in regulatory science; JSM is a professor expert in disability studies and social policy). At a later stage in the write-up of the results, two workshop participants were invited to critically review the developing manuscript and provide feedback in view of their respective experience and expertise: author EK who had taken part in the Australian workshop is a linguist and researcher on the topic of the information available for parents of deaf children, and author BMO participated in the UK workshop and is Public Involvement Manager for the Health Research Authority, UK. Both have personal experience of hearing loss, either having hearing loss themselves or as a family member of someone with hearing loss.

## Results

### Participants

Twenty-nine participants aged between 18 and 79 years joined the workshops (Table 1). 24.1% of participants had a self-reported hearing loss and 13.8% used sign language. Overall, eight relevant parties (i.e. groups who would be personally or professionally affected by the topic of this study) were represented amongst the workshop participants. Eleven participants selected the category ‘other’ to add detail about their specific role or a previous role that they held and that is relevant to the topic of this study (e.g. a role within a consumer representative group or a professional association).

**Table 1:** Demographic characteristics of workshop participants

	**Total (%) (*N* = 29)**
**Gender**	
Woman	18 (62.1%)
Man	10 (34.5%)
Non-binary	0 (0.0%)
Prefer not to say	1 (3.4%)
**Age**	
18–29	1 (3.4%)
30–39	3 (10.3%)
40–49	12 (41.4%)
50–59	9 (31.0%)
60–69	3 (10.3%)
70–79	1 (3.4%)
80 +	0 (0.0%)
**Ethnicity**^**a**^	
White (any background)	28 (96.6%)
Other or not reported	1 (3.4%)
**Relevant/interested parties represented**^**b**^	
Adult with hearing loss	7 (22.6%)
Family member of person with hearing loss	9 (29.0%)
Hearing health professional	9 (29.9%)
Hearing device manufacturer employee	4 (12.0%)
Involved in the development of hearing-related pharmacological therapies	0 (0.0%)
Involved in the development of hearing health-related policies and regulations	8 (25.8%)
Hearing health researcher	9 (29.0%)
Other (holds at least another role relevant to the field of hearing and deafness, such as chairperson for a hearing-related organisation, or previously held another relevant role)	11 (35.5%)
**Reported having hearing loss**	
Yes	7 (24.1%)
No	22 (75.9%)
**Reported using a hearing device**	
Yes	4 (13.8%)
No	25 (86.2%)
**Types of hearing device used**^**a**^	
Hearing aid	4 (13.8%)
Cochlear implant	2 (6.9%)
Bone Anchored Hearing Aid (BAHA)	0 (0.0%)
Other hearing implant	0 (0.0%)
Hearable	0 (0.0%)
FM system	1 (3.4%)
Personal amplifier	0 (0.0%)
Other	0 (0.0%)
Do not use a hearing device	21 (72.4%)
**Reported using sign language**	
British Sign Language (BSL)	0 (0.0%)
Auslan	4 (13.8%)
Use a different sign language	0 (0.0%)
Do not use sign language	25 (86.2)

^a^Participants were asked to identify their ethnic group. The responses were then collated according to UK Census List of Ethnic Groups [24]

^b^Participants could select multiple groups therefore totals exceed 100%

### Stakeholders’ views on regulation and innovation in the hearing health sector

The analysis of participants’ views covered three broad themes: (1) conceptualising regulation and innovation; (2) the need for ethical congruence between innovation, regulation and societal values; and (3) shifting roles and responsibilities (see Table 2).

**Table 2:** Themes and subthemes describing stakeholder perspectives of innovation and regulation in hearing health care

Theme	Subtheme	Exemplar quote
Conceptualising regulation and innovation	1a. Innovation and regulation is about more than technology	‘*With the regulation for hearing, is there regulation on the whole person, I guess, if that makes sense? Their language development, as a person are they involved in their community and their family life, are they able to socialise within their community? So taking the person as a whole, I don’t know how you can regulate one without considering the rest.*’ (participant 1, Australia)
	1b. Quality and risk: drivers of innovation and regulation	‘*For as long as I can remember, we have been trying to move away from the medical model in audiology more to patient-centred care and giving people the tools to be able to manage their hearing and their hearing health. So technology can only help with that. But we do need to be careful to not put pressure on people who are not able to [use technology] or have too many expectations that everybody is going to be able to manage that through the technology.*’ (participant 12, UK)
	1c. Perception of regulation as context dependent	‘*[Regulation] addresses the harm after it has occurred rather than before it occurs …. but it quite commonly comes back to the underlying human who is performing their everyday services or receiving services.*’ (participant 13, Australia)
Ethical congruence between innovation, regulation and societal values	2a. A desire for consumer involvement at every stage of innovation and regulation	‘*that’s what’s been missing, within Deaf Community [research] in the past—users have not been involved in the design*’ (participant 11, UK)
	2b. A desire for innovation and regulation to tackle perceptions of stigma	‘*I almost feel that making HA available from manufacturers like Apple (airpods) makes them more socially-acceptable*’ (anonymous comment written during the UK workshop)
	2c. A desire for inclusive, accessible and ethical hearing health care	‘*One thing that can be kind of easily regulated is just the kind of the way, the language that is used in this space to talk about people and their lives and their hearing loss. It is obviously an issue that is very hurtful in the way that [hearing loss] is represented.*’ (participant 3, Australia)
	2d. A desire for fair and trustworthy business practices	‘*With OTC and a lower cost of entry this is likely to get worse as potential “cowboys” enter the market for a quick buck.*’ (anonymous comment written during the Australian workshop)
Shifting roles and responsibilities	3a. Shifting the responsibility to consumers	‘*…there is a blurring of what the individual is responsible for doing and what is the role of the hearing healthcare professional. Individuals may also lack knowledge /understanding of all the options available to them and in what circumstances they should/could be used.*’ (anonymous comment written during the UK workshop)
	3b. Shifting the responsibility to product manufacturers	‘*Our patients are used to sharing their information with their clinicians, and that’s sort of part of the deal. Patients know it is going to happen but obviously now we are moving into the world where manufacturers have a much more direct relationship with patients.*’ (participant 6, UK)
	3c. Navigating the dominance of hearing health manufacturers	‘*…big providers having their own chain of hearing clinics, so go to [specific clinic name], for example, there is a particular brand that they only use, nothing else, consumers and most Australians don’t understand that. They don’t see that.*’ (participant 10, Australia)

The three identified themes converged around a central, organising understanding that saw effective regulation and innovation within the hearing health sector as dependent on a holistic approach that places humans at its centre and requires the dynamic involvement of a range of individuals and organisations. The three themes are discussed further below, supported by verbatim quotes from participants. Because several participants in this study were part of more than one stakeholder group (hypothetical example: a researcher in the field who also has hearing loss and has a family member with deafness), the specific roles of the participants are not provided with the quotes. Not providing the specific roles increases the anonymity of the participants. When comments were provided in a written form only (Padlet discussion boards), the researchers were also unable to identify the authors of the comments. These instances are noted as *anonymous comment written during the Australian (or UK) workshop*. Any clarification added to the quotes by the researchers is noted with brackets within the quote.

### Theme 1: conceptualising innovation and regulation in hearing health care

#### 1a. Innovation and regulation is about more than technology

Participants discussed a range of innovations during the workshops. These discussions highlighted the depth and breadth of innovation taking place in the hearing health sector. Discussions about technological innovations included hearing devices (e.g. hearing aids and cochlear implants) and other technologies (e.g. apps) to support remote monitoring and self-management of devices and rehabilitation. In light of the rapid pace of technological innovation in hearing health, there was considerable consensus amongst all participants that a ‘device-centric’ focus to innovation and regulation risked side-lining non-technological innovations of potential benefit to consumers. Innovation in service delivery reflected new models of care such as ‘direct-to-consumer’ (DTC; where hearing-related products and services are provided directly from the device manufacturer to the consumer without the involvement of a traditional hearing health care professional such as an audiologist) was discussed. Pharmacological therapies and other biotechnologies were also discussed in the context of hearing restoration and genetic testing for hearing loss. Participants highlighted the human aspects that are embedded within the hearing health sector, beyond the technology, and the importance of centring discussions about innovation and regulation within a holistic person-centred approach.

*With the regulation for hearing, is there regulation on the whole person, I guess, if that makes sense? Their language development, as a person are they involved in their community and their family life? Are they able to socialise within their community? So taking the person as a whole, I don’t know how you can regulate one without considering the rest.* (participant 1, Australia)

Australian participants suggested that an emphasis on technology over other interventions (e.g. aural rehabilitation and counselling) limited how innovation and regulation were conceptualised in the hearing sector.

*The misunderstanding that a device alone is not enough. Another way to consider this, is a lack of value placed on the importance and nuances of hearing loss and communication behaviours.* (anonymous comment written during the Australian workshop)

*We need to be really cautious of that [push on device sales] becoming an even bigger problem with over-the-counter hearing aids and future technology. When people have hearing impairment, technology is not necessarily, or devices are not necessarily, the answer.* (participant 11, Australia)

A UK participant also commented on the growing ‘digital divide’ that has developed: with millions of people in developed countries not being able to use digital technologies due to a lack of support and training. This emphasises the need for innovation and regulation to encompass hearing care beyond hearing technologies.

#### 1b. Quality and risk: drivers of innovation and regulation

Participants from both countries suggested regulation and innovation shared the common goal of ensuring quality and minimising risk. They viewed quality and risk to be inversely related in the sense that high-quality hearing health care was presumed to minimise risk, which participants defined implicitly as potential for harm. Quality and risk therefore were lenses through which innovation and regulation were viewed, contextualised and understood by participants.

Participants viewed quality as a cross-cutting, multi-dimensional construct. Quality was discussed in the context of (1) the effectiveness, safety and suitability of interventions (including devices and other technologies, aural rehabilitation and pharmacological therapies); (2) the delivery of services (e.g. timeliness, patient-centredness); and (3) information sharing (e.g. accuracy and reach). Some participants were optimistic, describing technological innovation as ‘positive’ and ‘exciting’, with potential to improve patient outcomes through greater empowerment. Positive outcomes, however, were perceived as not only related to quality technology, but to ensuring the suitability of different technologies for different individuals.

*For as long as I can remember, we have been trying to move away from the medical model in audiology more to patient-centred care and giving people the tools to be able to manage their hearing and their hearing health. So technology can only help with that. But we do need to be careful to not put pressure on people who are not able to [use technology] or have too many expectations that everybody is going to be able to manage that through the technology.* (participant 12, UK)

Participants highlighted safety concerns in relation to innovation, which would need to be addressed to ensure quality. Self-fitting devices were suggested to pose risks including potential for further hearing damage arising from overamplification or risks of missed or delayed diagnoses if hearing was not evaluated by a trained hearing healthcare professional (e.g. audiologist, otolaryngologist). Participants further highlighted risks related to the efficacy and safety of devices purchased online from unregulated vendors.

*...patients buy medical devices online for many reasons - they don’t get advice from the clinicians, go online, [devices] have fraudulent CE marks attached to them [CE marks aim to attest compliance to European standards; CE*=*Conformité Européenne]...it’s just a horrendous situation that we find ourselves in.* (participant 9, UK)

Participants discussed how the promise of new treatments for hearing loss could also impact on the quality of outcomes and could pose a further risk if consumers opted to wait for new treatments not yet on the market or available to healthcare providers and consumers (e.g. cellular therapies to restore hearing loss). The majority viewed timeliness as a mark of quality. One participant commented in the chat ‘the best treatment [for hearing loss] is the treatment that is available now’. Other participants highlighted potential risks to wellbeing and cognitive health associated with delayed intervention for hearing loss. They stated that most consumers lacked awareness of these risks, attributing lack of awareness to the inconsistent quality and effectiveness of public health messaging about hearing loss. Lack of reliable, high-quality information was also viewed as perpetuating misconceptions about the availability and quality of publicly funded hearing healthcare services (such as the UK’s National Health Service; participant 1, UK).

*I think we need to be mindful that actually there is a general lack of public awareness .... It’s [hearing loss] considered an invisible disability - doesn’t matter much, not life threatening, no-one will die of hearing loss. [Yet] we know it has a huge impact. It does matter. So it’s getting that across because you can bet everybody knows somebody that’s had a hearing loss* (participant 11, UK)

#### 1c. Perception of regulation is context dependent

Perception of regulation varied by country and by interested or affected group. In Australia, some participants expressed a degree of scepticism towards regulation, asking ‘who regulates the regulators?’ (anonymous comment written during the Australian workshop). Limitations to regulation—such as the retrospective application of regulation to innovation—were also highlighted. Some participants stressed the point that regulation is only ‘one tool in the toolbox’ (participant 13, Australia) for ensuring quality and minimising risk, noting also that successful regulation is contingent upon its implementation by human actors.

*[Regulation] addresses the harm after it has occurred rather than before it occurs .... but it quite commonly comes back to the underlying human who is performing their everyday services or receiving services.* (participant 13, Australia)

In the Australian context, some participants saw the need for greater professional regulation, since audiology and audiometry are currently self-regulated professions and privately funded hearing services can be offered without professional association membership.

*Audiology is currently NOT a registered profession under AHPRA [the Australia Health Practitioner Regulation Agency]. Regulating and allowing for registration of our profession would provide a better framework for hearing care (hence removing commissions in hearing aid sales).* (anonymous comment written during the Australian workshop)

*I don’t feel there is near enough regulation on who can prescribe hearing aids & the accountability of hearing aid suppliers.* (anonymous comment written during the Australian workshop)

While the UK professional regulation framework is different to Australia’s, its effectiveness was also questioned:

*Regulation of practitioners remains variable with three different routes for Audiology professionals 2 being statutory and 1 voluntary, hence the workforce is arguably unregulated.* (anonymous comment written during the UK workshop)

Overall, UK participants viewed current regulation positively, but considered further work essential for regulators to ensure quality and minimise risk. Participants considered regulation critical in ensuring safe practices around data capture, storage, sharing and use, particularly regarding personal data. Participants agreed that data should be used to facilitate and not hinder consumer benefit. It was suggested that fear was a key driver to the hearing sector’s response to data sharing and use.

*We need to tackle fear of data ..... it’s making sure we are open, transparent, honest on who uses what data where and being really clear about that. Making sure we are clear about that.* (participant 11, UK)

Concerns raised relating to data management practices included (1) direct industry interaction with consumers; (2) complexity of data capture, storage, sharing and use in a context spanning healthcare, medical devices, non-medical devices and other technologies; and (3) access to transparent information about how the data would be used. Technological innovations such as data logging—whereby a hearing device records and stores information relating to device use (e.g. types of acoustic situations a user encounters, frequency of device use, location of device)—were a specific concern. Participants suggested many consumers did not have a clear understanding of what data were being collected and how these data were being utilised. Some expressed worries that lack of clear communication and thus understanding could potentially lead to legal claims in the future (participant 5, UK). The sharing of personal and clinical data with device manufacturers, through the outsourcing of services traditionally delivered by healthcare professionals (e.g. device maintenance and repair), was also a source of unease. Others worried that consumers may be put off by the complex nature of data storage and ultimately decline to engage with technology and other interventions, potentially resulting in missed opportunities to improve care and support, and communication outcomes.

*...patients should be able to sign up to what data is collected, what it’s used for. I think the difficulty is making it simple enough because it is very complex ..... it needs to be clear what you are signing up to. So often in an appointment you are given this “Oh sign this for our records” and a) you don’t read it, b) if you did read it, it’s not simple enough for a lot of people to understand. I am guilty of not signing up for apps and doing lots of things [because] I don’t like my data being used. I suspect there are a lot of people like me, and we may be missing out on something that may be tremendously valuable because we are put off by pages and pages of things you have to sign.* (participant 13, UK)

### Theme 2: the need for ethical congruence between innovation, regulation and societal values

The groups represented at the workshops appeared to share a number of common values. These values are a set of contextualising factors which participants used to define quality and risk in the context of innovation and regulation. Participants hoped that developments within hearing health care would be congruent with these values.

#### 2a. A desire for consumer involvement at every stage of innovation and regulation

Participants in both countries expressed strong preferences for consumer and public involvement in regulation, innovation and the research underpinning innovation. Consumer engagement was described as critical to ensuring relevance and quality, while minimising risk. UK participants described recent initiatives to embed consumer involvement across regulation and research. One participant expressed their approval of these efforts, noting ‘that’s what’s been missing, within Deaf Community [research] in the past—users have not been involved in the design’ (participant 1, UK). An Australian participant similarly articulated the importance of consumer involvement in the following comment: ‘*Innovation may not always reflect the difficulties or needs that people experience in real life*’ (anonymous comment written during the Australian workshop). Participants emphasised that when designing new interventions, it would be essential to involve consumers at every stage of the product design lifecycle, including how information describing innovation is written and disseminated.

*...go back to that patient after the procedure and say, we said we were going to do x, y or z, did x, y or z actually happen? So having more patient involvement in constructing clinical evaluations is something as a regulator we are putting forward.* (participant 9, UK)

*The other really strong point that is coming out is about ‘nothing about us without us’. So I think that is a really such a clear point, but actually we do it, we do it quite poorly at the moment. We need to really understand the perspectives of people who are - the deaf community, people with hearing loss, people with hearing impairment. We need to be open to all the different perspectives in this debate, and it is not an easy thing,* [to] *bring together all of those different perspectives, so we need to know that it is not just everyone agrees, and we all know where to go. It is an iterative process that is going to keep changing as the technology changes as well.* (researcher JSM summarising the main insight expressed by the workshop participants in Australia)

To achieve meaningful consumer and public involvement in hearing health care, participants from both countries suggested that a paradigm shift away from a medical, expert-driven model of care as well as away from care models driven by a profit-oriented approach. Adopting a social model of disability (which centres on making societies, services and places more accessible for everyone instead of trying make people with disability ‘fit in’ within inaccessible environments [25]) was suggested to help achieve this shift as it requires meaningful involvement from individuals with disability.

#### 2b. A desire for innovation and regulation to tackle perceptions of stigma

Innovation and regulation in hearing care were viewed as opportunities to address perceived stigma associated with hearing loss which often compounds the stigma of ageing. The stigmas of hearing loss and ageing, together with the conception that hearing loss is part of the normal ageing process, were viewed as significant barriers to hearing care. That is, individuals may delay hearing care or access incomplete communication support because of the negative perception they or their family have about hearing loss, hearing device, sign language or ageing.

*Hearing aids get such a bad rap. I fitted hearing aids to a 90 something year old - she was so worried they would make her look old.* (anonymous comment written during the UK workshop)

Perceived stigmas can lead to denying, hiding or normalising increased hearing difficulties instead of exploring potential communication support options [26]. The availability of new and more streamlined forms of technologies and services such as self-management apps, access to direct-to-consumers or over-the-counter (OTC) hearing aids and the use of mainstream consumer technologies (i.e. Apple Airpods or other hearables) to manage hearing loss were innovations that participants considered could reduce stigma by promoting access to care outside of traditional clinical settings.

*I almost feel that making HA available from manufacturers like Apple (airpods) makes them more socially acceptable.* (anonymous comment written during the UK workshop)

By considering interventions beyond traditional hearing technologies and situating hearing health innovation and regulation within a social model of disability, participants proposed that digital innovations for managing hearing loss, deafness and hearing care could be less stigmatised. The value of education in this respect was further highlighted, some viewing education as a way to foster ‘*…more awareness around hearing loss, take it out of the darkness, more conversations about it to remove the stigma*’ (anonymous comment written during the UK workshop). A similar point was made in relation to the need for normalising discussions around sign languages, to broaden communication opportunities:

*I grew up as a hearing aid user for nearly all my whole life, so from 15 months of age, and at no point was I exposed to Auslan. I discovered Auslan as an adult and went “Wow, this is brilliant”. If we could look at regulation that would ensure that every child who was hard of hearing had the option to consider Auslan as one of the benefits or one of the tools that they can use in their life and that culture that comes with that as well, and that acceptance of being somebody who is hard of hearing and not fixing it but actually gaining from having a language like Auslan.* (participant 16, Australia)

#### 2c. A desire for holistic, inclusive, accessible and ethical hearing healthcare

Participants suggested that innovation and regulation in hearing health care provided an opportunity to promote equity, diversity and inclusivity. They recognised that culture plays a central role in ensuring equitable and inclusive hearing health care and that hearing care professionals have a responsibility to actively identify and address sources of bias when striving for quality and minimising risk.

*I was involved in one group talking about Aboriginal and Torres Strait Islander people with hearing loss and it was interesting in that group they said, “No, I don’t want to be talking about that topic. That’s a topic for the indigenous community”. I thought that was very interesting that because it’s okay for hearing people to talk about deaf people, but it’s not okay for non-indigenous people to talk about indigenous people. So it is something to keep in the back of our mind as we move forward in discussions to make sure that deaf people are recognised as people too…* (participant 1, Australia)

Regulating the language used to communicate hearing health information was suggested to promote inclusive and ethical care:

*One thing that can be kind of easily regulated is just the kind of the way, the language that is used in this space to talk about people and their lives and their hearing loss. It is obviously an issue that is very hurtful in the way that [hearing loss] is represented.* (participant 3, Australia)

The cost of innovation was identified by some participants as a barrier to consumers accessing the latest hearing technologies. Cost barriers were therefore suggested to restrict access to innovation and limit inclusivity. Participants also discussed worries associated with the sustainability of publicly funded innovative services and interventions, in particular when these services are provided for free by hearing device manufacturers (e.g. aural rehabilitation apps). They described an ethical dilemma in so far as they are eager to offer these innovative services, but need to balance this against a risk for future harm to patients should funding be cut or currently freely available services be associated with a cost in the longer term.

*One of the things that ……I think is really important is that currently a number of these systems that are available through the manufacturers are free. And we’re all capitalising on those. From that we can do some quite nice cost-benefit exercises as to why it would really be useful for us to introduce these things. But I think there is a massive risk here that this is not going to be free forever because these things are very costly to keep updated in terms of applications.* (participant 5, UK)

As public services grapple with the challenges of increasing workloads and decreasing budgets, there was a concern amongst healthcare professionals that cost could hinder innovation with organisations prioritising the delivery of essential services. In such situations, participants worried about the ethical implications if innovations fail to reach all intended beneficiaries. Participants endorsed an ethical approach to innovation and regulation, which respects and supports autonomous decisions and promotes nonmaleficence, beneficence and justice [27].

#### 2d. A desire for fair and trustworthy business practices

Hearing care in the UK and Australia are delivered by a range of publicly funded and for-profit organisations and individuals. It was clear from participants that discussions about innovation and regulation in the hearing health sector must consider business-related aspects and market factors. In particular, participants discussed the importance of having transparent systems to promote fairness and trust within hearing health care. Participants discussed concepts of ‘endorsement’, ‘warranties’, ‘professional standards’ and ‘national registries’ to report on efficacy and safety events, as ways to promote transparency and trust in the quality of products, services and information.

*People are constantly seeking information/products from places they trust, that could be big brands, patient organisations or the NHS. In order for people to feel like they are getting the best product/service, they may look for some kind of endorsement from these sources.* (anonymous comment written during the UK workshop)

Participants discussed how potential conflicts of interest could impact on the quality of services. To promote trust in the sector, transparency about sales targets and financial remuneration (e.g. benefits, commissions) received by a provider as a result of a ‘sale’ (Australian participant) was considered important, if not essential.

There were also concerns related to several hearing device brands clustering within a small number of ‘parent companies’:

*Are the Big 5 [five largest hearing device firms] doing the best/right thing […] with consumer brands? - This doesn’t create more competition in the market, it siloes it.* (anonymous comment written during the Australian workshop)

Similarly, participants raised efficacy and safety risks that could come with consumers making purchases through non-regulated avenues, stating that ‘…*the role of regulator in [addressing the problem of] counterfeit devices also needs to be communicated*’ (UK participant). Participants worried that current innovation and regulation may contribute to the growth of opportunistic businesses that may not be able to offer quality care. This was more salient with the Australian compared to the UK participants.

*With OTC and a lower cost of entry this is likely to get worse as potential “cowboys” enter the market for a quick buck.* (anonymous comment written during the Australian workshop)

Moreover, being unable to confirm the quality of devices purchased online impacts the extent of services hearing care professionals can provide:

*Can that money be better spent getting a brand new [device] where it comes with a three-year warranty, and we have to make sure that you’re satisfied with it. If they’re paying for a fitting fee, it is a fit. I can’t guarantee whether it will be amazing. We will do the verification, but you won’t get a warranty with it because we don’t know where you have purchased it from.* (participant 15, Australia)

A number of participants also discussed the importance of having effective regulation to limit inadequate advertising practices in order to create trust in hearing care. Inadequate advertising practices were discussed in relation to device manufacturers, clinical services and social media using targeted marketing. These practices were raised as a particular concern for Australian participants. For example:

*There is often a significant gap between advertised claims and fulfilment in use. […] This needs clear and VISIBLE policing to ensure only quality products and prudent advertising claims* (anonymous comment written during the Australian workshop)

*The industry is nowhere near regulated enough at the moment and not enough basic benchmarks. Too much advertising that claims that once you have a hearing device or two all will be just dandy. This pretty much false advertising* (anonymous comment written during the Australian workshop)

*I’m connected with a deafness information page on [social media provider], so these are the ads that get pushed to my feed on [tech company] News. It is quite frustrating because it happens all the time. There is just no way that I can see how to stop that type of directed targeted marketing. I wonder how many other people feel pressured by [this] constant stream of promotion.* (participant 1, Australia)

### Theme 3: shifting roles and responsibilities

Participants conceptualised the hearing health sector as a network of organisations (e.g. consumer groups, private and public clinics, technology developers and manufacturers, research and education organisations, regulatory bodies) and individuals (e.g. consumers, clinicians, engineers, managers, researchers) having different knowledge, roles and interests. Workshop discussions suggested that these roles, interests and knowledge were in a state of flux that was fuelled, in part, by innovation and regulation.

#### 3a. Shifting the responsibility to consumers

In particular, innovation was perceived as a driver for a loss of professional accountability, with greater responsibility for care being handed from clinicians to consumers, without the underpinning clinical knowledge. This was perceived as a risk that was not yet addressed through existing regulation.

*With the introduction of new technologies (e.g., OTC, remote fitting/adjustment) there is a blurring of what the individual is responsible for doing and what is the role of the hearing healthcare professional. Individuals may also lack knowledge/understanding of all the options available to them and in what circumstances they should/could be used.* (anonymous comment written during the UK workshop)

A shift in roles and responsibilities was perceived as inadvertently complicating access to hearing products and services. In Australia, decision-makers for one of the main hearing care funding schemes seemed to have altered the expected support role audiologists and audiometrists would take with their clients:

*the NDIS [Australian National Disability Insurance Scheme] will not communicate with providers [,] which creates a system that is difficult to navigate for participants and providers who are trying to help them.* (anonymous comment written during the Australian workshop)

#### 3b. Shifting the responsibility to product manufacturers

Participants also discussed the gradual shift from clinician-led hearing services towards a more direct-to-consumer service model offered by, or in collaboration with, product manufacturers. This discussion highlighted a lack of clarity and transparency about the regulation already in place to manage accountability and the potential risks related to consumer data shared between organisations.

*Our patients are used to sharing their information with their clinicians, and that’s sort of part of the deal. Patients know it is going to happen but obviously now we are moving into the world where manufacturers have a much more direct relationship with patients and we are having to share patient information in a limited way directly with manufacturers so for example patients can get equipment delivered by courier direct from manufacturers using the apps and remote care services.* (participant 6, UK)

The possibility of clinicians losing part of their professional responsibilities was raised as a potential de-skilling of the workforce or an underutilisation of the full range of skills audiologists are trained to use. Solutions such as the unbundling of services (i.e. charging for items or services separately rather than as part of a device-rehabilitation service package that combines the purchase of devices) were suggested in Australia as key ‘*…for flexibility and to make clear the value (and cost) of [health care professional] services*’. In the UK, greater regulation of purchases made directly from manufacturers was suggested, together with clear information about responsibilities within hearing care services. As a UK participant asked: *what is the role or most appropriate role for clinicians in this environment of rapid technological change?*

#### 3c. Navigating the dominance of hearing health manufacturers

Throughout both the UK and the Australian workshops, participants raised concerns related to the perceived current dominance of hearing device manufacturers and the influence of corporate entities/organisations on hearing health care decisions. Participants suggested that the ‘*…lack of regulation in the Audiology health care sector allows big brands to dominate health care creating an even greater device-centric system.*’ While many agreed that ensuring access to quality and appropriate technologies is a core component of hearing services, there was a perceived imbalance of power across organisations and individuals, accompanied by a perceived lack of options and transparency for consumers. Participants highlighted that hearing device companies may also own hearing services clinics under a different name, sometimes unbeknownst to clients.

*I agree with the comment about the big providers having their own chain of hearing clinics, so go to [specific clinic name], for example, there is a particular brand that they only use, nothing else, consumers and most Australians don’t understand that. They don’t see that.* (participant 10, Australia)

*For cochlear implants, we now only have three manufacturers which is not ideal for healthy competition.* (participant 6, UK)

Overall, participants in this study described their wish for a whole person approach to quality hearing health care. They reflected on the dynamic interplay between innovation and regulation and how this interplay influences the roles and responsibilities within the field, which impacts on their perception of quality care. They further discussed the importance for ethical values to guide all phases of innovation and regulation.

### Current initiatives

As participants discussed issues related to the three themes of this article, they also referred to recent positive initiatives in each country. For example, during the UK workshop, participants shared several web links including advice for buying medical devices online [28], recent initiatives to engage patients and the public in health product regulation [29], and a charity that aims to unite the UK’s health and care data [30]. In Australia, a participant compared the workshop discussion to the work led by the Hearing Health Sector Committee (now the Hearing Health Sector Alliance), which led to a Hearing Health Roadmap [31] and governmental funding for subsequent initiatives. While more work is clearly needed to better align valued regulation and services with new and coming hearing health innovation, several encouraging initiatives are underway.

## Discussion

This study explored the perspectives of multiple informed groups on the topic of regulation and innovation in hearing healthcare. In conceptualising this topic (theme 1), participants agreed that innovation and regulation need to encompass all aspects of a person’s life, beyond the use of technology. The data generated in this study also highlighted how regulation and innovation were driven by perceptions of quality and risk, and that the perceived value of any regulation is context-dependent, i.e. not all regulations are implemented in ways that are perceived as valuable for all consumers. Participants expressed a desire for regulation and innovation in hearing health care to be ethically congruent with societal values (theme 2). As such, innovation and regulation should involve consumers at every stage and promote inclusive and accessible hearing care. It was also suggested that more could be done in terms of regulation and innovation to promote fair and trustworthy business practices, as well as to reduce perceptions of stigma. Finally, participants discussed how current innovation and regulation are driving a shift in roles and responsibilities (theme 3) with traditional clinical service responsibilities increasingly becoming the remit of consumers and product manufacturers. While this shift can promote greater accessibility and consumer empowerment, it also risks creating a more competitive system that promotes individual products instead of holistic hearing health and communication support. This shift was framed with a perception that the current system was already difficult to navigate and tended to promote the interests of product developers over those of other groups, including consumers.

Several of the subthemes discussed in this study have been central issues within health services research for at least 60 years highlighting the ongoing dynamic interplay between regulation, innovation and quality care [e.g. 32–35]. For example, the participants’ views in the present study about the need for more holistic (and not merely device-centric) care echo issues and concerns raised by White et al. who noted that in comparison to interest in technology, ‘*Human and personal values have been relegated to an inferior status*’ [36]. Indeed there is a vast literature illustrating the importance participants in other health contexts have ascribed to adopting a whole-person multi-dimensional perspective beyond technology [36], the central role of consumer involvement in all phases of health care innovation and regulation [33], and the desire for inclusive, accessible and ethical health care [32]. While the prominence of these issues within our discussions could suggest that they are now well-anchored within the Australian and UK’s expectations of health care, their prevalence within the data also implies that realising these goals remains a work in progress. It is likely that participants wanted to re-assert the importance of such values to ensure that these would not be dismissed with further innovation and regulation. The prominence of these concepts in our results may also reflect a gap between the desire for such values to drive health systems and their difficult implementation in practice. A relative neglect or lag in implementing more ethically oriented markers of quality (e.g. patient-centredness, equity) has been noted elsewhere, including in a recent scoping review of the USA hospitals’ quality performance assessments [37]. Such research suggests that long-held quality values are still not included within routine assessment of quality care, in spite of recent emphasis on the importance of value-based care, defined as ‘equitable, sustainable and transparent use of the available resources to achieve better outcomes and experiences for every person’ [38]. Value-based care has been suggested in response to increased demands on healthcare systems, driven in part by the continuous stream of innovation and new technologies [39]. Regulatory organisations and processes, which exist to support the implementation of quality care, must therefore also be dynamic and continuously adapt to ongoing innovation.

While core concepts discussed by our participants mirror long-held discussions related to innovation and regulation within health care, our findings add to this body of literature by underlining key contemporary challenges. In particular, (1) data capture and transfer between medical and lifestyle technologies has further blurred boundaries between what is considered medical or general consumer products and services, and using both types of innovation together must be considered as part of care and as part of data privacy regulation; (2) holistic care for deaf and hard-of-hearing individuals, which combines a flexible range of communication and technology options, does not align with the current, mainly siloed, organisation and funding of hearing services and products; (3) digitisation and automation have promoted a broadening of organisations and individuals that are part of the ‘hearing health sector’ and greater involvement of a range of private enterprises. While this broadening is welcomed as it is already improving access to care and de-stigmatising hearing technologies, it also creates more opportunities for unchecked products, services and information to reach consumers; and (4) transferring hearing health responsibility to consumers with varying health and technological literacy levels risks increasing health disparities [as discussed in 40, 41]. While more affordable and accessible technology is expected to reduce disparities, it could also lead to a reduction in accessing effective support for those who may need it the most. This includes support for choosing and learning how to use the technology or selecting and implementing effective alternative or complementary approaches to support communication [42]. These four challenges are reflected in participants’ reports that current products and service providers are not adequately regulated, in their impression of an over-reliance on technology, their concerns about an inappropriate dominance of device manufacturers over other relevant parties in the field, and a worry that an increased rate of innovation will make it more difficult to ensure trustworthy business practices and quality care. Similar challenges would likely be found in most fields of health care influenced by digital innovation.

Overall participants centred their reflection on the complexities of organising valuable services for deaf and hard-of-hearing individuals. The majority agreed that hearing health innovation should first and foremost benefit the holistic communication experiences of the person and be congruent with human-centred values, such as equity, diversity and inclusivity. Participants acknowledged the complexities related to achieving valued regulation, due in part to the diversity of people and organisational structures involved in the sector, but also to the dynamic nature of innovation and its influence on roles and responsibilities. Based on the findings of this study, we collated a list of recommendations (Table 3) to support the development of future innovation and regulation in the hearing health sector. We acknowledge that some of these recommendations have already been raised in other reports or are already promoted within a number of organisations. Our findings, however, suggest that more needs to be done to implement such recommendations at scale. Many of these recommendations are also directly relevant to other fields of health care that embed rapid innovation and digital technologies.

**Table 3:** Recommendations informed by the findings of this study, for valuable future innovation and regulation in the hearing health sector, or applicable broadly across health care

1	Health care innovation and regulation should actively promote equity, diversity and inclusivity, in addition to safety and effectiveness
2	Members of the target patient population and the public should be involved in the co-design of relevant regulation and regulatory processes, as well as across all phases of innovation development
3	Regulation should take a prospective and preventative view, through horizon scanning of the likely requirements for future regulation, developed in tandem with innovation
4	Innovation and regulation should ensure transparent and safe data management and sharing practices *across the health, disability and care sectors*, including public and private entities, and take into consideration consumer products (such as smartphones) that enhance the functionalities of connected health devices (such as hearing aids). Because consumer products can now gather extensive personal information that can be more revealing than what is commonly collected for health service purposes, regulatory agencies should review the boundaries of what might be considered a health product and service
5	Impartial, complete and evidence-based information describing emerging and new innovations should be freely available in accessible formats for patients and the public. This information should be collated and endorsed by independent groups with relevant expertise who can explicitly manage potential risks of undue influence from parties with financial ties to the product or service
6	An impartial review of professional regulations appears required, to ensure these are effective and fit-for-purpose considering new and coming technology, service and information provision. These should also clarify the roles, responsibilities and accountability of all interested and affected parties. This is particularly relevant for consumers who may need clinical support after purchasing, or to purchase, a general consumer product
7	Hearing health care should be conceptualised holistically, integrating an individual’s life-long hearing health and communication needs beyond the provision of hearing devices. Complementary, alternative and evidence-based approaches must be supported if gold-standard technologies add to an individual’ burden, are inaccessible or provide limited benefits to that individual
8	Reviewing and implementing effective business policies and practices is required to safeguard and promote safety, fairness and trust within the broader hearing health care market

Individuals and organisations with a range of interests and perspectives need to work together for ongoing refinement and structuring of effective support for our societies’ wellbeing [43]. Because new policy and regulation often seem to fall short of delivering the intended benefits, it is understandable that discussions about regulation can be met with cynicism [44]. Regulation, however, must evolve as new knowledge becomes available and by learning from previously trialled regulatory decisions [44]. As presented in Hudson [43], several UK initiatives have built on one another to better integrate care across services and to instil more robust ethical behaviour within all levels of care provision. An example is the Committee on Standards in Public Life created in 1994 that led to developing the ‘Nolan Principles’ to address confidence in political systems [45]. In Australia, the 2018 National Integrity Commission Bill and the establishment of the National Disability Insurance Scheme in 2016 are other examples of new regulation and structures with objectives that align with the desires discussed by participants in this study, in relation to ethically congruent regulation and innovation in hearing health care. Often, however, little evidence of practical improvement is reported after such initiatives, or further complexities are exposed through the changes [46, 47]. This means that a different approach to developing and implementing recommendations towards valued integrated care is needed [43, 48]. When care structures relate to a common chronic health condition such as hearing loss and deafness, a condition that directly impacts the experience of communication and wellbeing and that is a breeding ground for digital innovation and market competition, an overarching holistic perspective must be taken. Without this overarching approach, narratives of hope through simple technological fixes can blur the ongoing and contextualised communication needs of deaf and hard-of-hearing individuals.

The findings of this study align with governmental directions such as the Australian Roadmap for Hearing Health [31] and the Australian Report of the independent review of the Hearing Services Program [49]. Those reports also highlight the need for more holistic services and increased consumer input within the development of products and services. Our findings, however, extend to the arrival of new digital products and service providers in the field, impacting the traditional role of audiologists, audiometrists and hearing device providers, with limited structures to ensure accountability beyond these professions. The concept of data privacy was also discussed beyond the data collected by clinics and researchers, now extending to data collected by apps, smartphones and device manufacturers. Digital connectivity opportunities now provide means for private enterprises that are not regulated within health and disability services to offer hearing products, services and information. Consumers can access these directly online without the knowledge of the potential risks involved or alternative care options.

### Strengths and limitations of this study

To the authors’ knowledge, this is the first qualitative study exploring the views of various relevant groups in relation to innovation and regulation in the hearing health sector, a sector with a long history of integrating digital health innovation within wearable devices. While other reports have provided recommendations based on stakeholder collaboration, no studies have explicitly sought to describe the ‘lay of the land’ and the perceived opportunities and risks from the perspective of stakeholders. We also believe that the complementarity of different knowledges and expertise of the co-authoring group is a strength of this article, with the contributions of the participants in this study benefiting from combining our respective perspectives. In addition, an early iteration of the thematic analysis was shared with workshop participants to confirm the interpretative validity of the findings [22]. This study further combines the views of relevant parties across countries with different regulation and service delivery models. The strength of this approach is it enabled the authors to examine the data for universals, enhancing transferability. Further work is needed to examine the findings across other countries and health systems.

The study also has limitations. We aimed to sample purposively for maximum variation for the variables of age, sex, communication modality and device use, as well as representation from different stakeholder groups. However, as most participants were White/Caucasian, the perspectives of different ethnic groups, largely minoritised populations in Australia and the UK, are not represented in the data. Nor was there representation from private dispensers or users of sign language as their primary communication mode in the UK. This study applied a reflexive and constructivist approach: an approach that openly acknowledges the way that researchers’ knowledge, experience and expertise can influence the way that data is gathered, interpreted or presented. For example, the researchers’ decision to invite additional authors with lived experience to the team, rather than representatives of device manufacturing firms, may have resulted in perspectives of people with lived experience being held in higher esteem than might have otherwise occurred. This said, all efforts were made to ensure all viewpoints were represented as faithfully as possible and several opportunities were offered to all participants to contribute their ideas (publicly or anonymously), before, during and after the workshop with this window being open until the last version of this manuscript was submitted. Research to explore the perspectives of informed groups not represented in the data is now needed. In future, studies will also be required to understand how equity, diversity and inclusivity considerations, as well as how an holistic conceptualisation of hearing health care, impact the latest innovation: the rapidly evolving field of cell and gene therapies for hearing loss [50].

## Conclusions

This qualitative study presents the collective perspectives of various interested groups on the topic of regulation and innovation in the hearing health sector. Adults with hearing loss, family members, hearing care professionals, regulators, industry representatives and policymakers regarded regulation and innovation to be essential vehicles for minimising risk and ensuring the quality of hearing technology, services and information. To be considered successful, both regulation and innovation should have active involvement from consumers; be ethical, equitable and inclusive; and promote holistic, fair and trustworthy practices. The findings highlight the interconnectedness of these factors and emphasise that valuable approaches to regulation and innovation in hearing healthcare must be based upon collaboration and shared responsibility. This study serves as a guide for policymakers, regulators and industry, urging them to prioritise inclusivity, transparency and fairness in their efforts to regulate and innovate within a rapidly changing healthcare system. Doing so will help to create a more responsive and patient-centred hearing health sector that ensures the well-being of individuals with hearing loss and their families.

## Supplementary Information

Supplementary Material 1.

Supplementary Material 2.

## Data Availability

Data described in this manuscript which have been collected by the research team during this study and that can be anonymised, can be made available upon reasonable request to the corresponding author and subject to research governance approvals.

## Abbreviations

BAHA: Bone Anchored Hearing Aid
BSL: British Sign Language
CE: Conformité Européenne
COREQ: COnsolidated criteria for REporting Qualitative research
DTC: Direct-to-consumer
OTC: Over-the-counter
NDIS: National Disability Insurance Scheme
UK: United Kingdom
