# Supplementary material for: A qualitative reflexive thematic analysis of innovation and regulation in hearing health care
Source: BMC Med. 2024 Sep 27;22:417. doi: 10.1186/s12916-024-03627-1 (PMC11438185; doi:10.1186/s12916-024-03627-1)
Supplement: Supplementary file 1 — Supplementary Material 1. [file 12916_2024_3627_MOESM1_ESM.pdf]

1st November, 2022

Dear Participant,

Thank you for agreeing to take part in the online stakeholder workshop on hearing innovation and regulation that will take place on Tuesday 8 November.

Included in this document are:

1. The formal online consent form and demographic survey **(needs to be completed before the meeting).** Click here to complete the form**:** **https://www.smartsurvey.co.uk/s/5PD7U3/**
2. Suggested pre-reading
3. Workshop agenda
4. Topic guide and scenarios for workshop discussion
5. Ground rules and guidance for this Zoom meeting
6. The participant information sheet about this study

Communication support
The online workshop will be live captioned and with Auslan interpreting. If you require additional communication support needs, please let us know at your earliest convenience.

On the day
If you need to contact us on the day, please email Isabelle Boisvert at isabelle.boisvert@sydney.edu.au or Jennifer Smith-Merry at jennifer.smith-merry@sydney.edu.au

Thank you and we look forward to seeing you online.

Yours sincerely,

Dr Isabelle Boisvert and Dr Sarah Hughes
co-Principal Investigators on behalf of the Research Team

Suggested workshop pre-reading

Advancing regulatory science in hearing care: stakeholder perspectives

In recent years, several articles and conference presentations have discussed changes that are happening in the hearing health sector. This includes automated online hearing tests and hearing advice, purchasing over-the-counter hearing aids that do not need a recommendation, at-home self-adjustment of hearing aids, connecting hearing aids to smart phones and smart-home systems, developing drugs as a treatment for hearing loss, or integrating electrodes within hearing devices that could measure heart beats, cognitive effort or fatigue. Companies such as Apple, Google, Facebook and Samsung have also shown interest in developing products to support people who have hearing loss.

Below are some recent articles about changes in the hearing health sector. We would be grateful if you could look through these articles in advance of the workshop as they will help to provide context for our conversations during the workshop:

https://idainstitute.com/fileadmin/user_upload/Future_Hearing_Journeys/Report_V1/index.html#/lessons/L1tC2RlkRxj0qKdNo4rWXIZnnlqtAv2T

https://www.hearsoundly.com/guides/best-ai-hearing-aids

https://hearingreview.com/inside-hearing/research/consensus-on-the-use-of-emerging-hearing-devices-and-service-delivery-models-in-the-uk

https://www.medicalnewstoday.com/articles/best-online-hearing-test#how-to-prepare

https://www.theguardian.com/society/2019/may/15/scientists-create-mind-controlled-hearing-aid

https://news.mit.edu/2022/frequency-therapeutics-hearing-regeneration-0329

Thank you!

Advancing Regulatory Perspectives in Hearing Healthcare
Stakeholder Workshop

Tuesday, 8th November 2022

Sydney/Canberra/Melbourne/Hobart: 3:00pm – 5:00pm
Adelaide: 2:30pm – 4:30pm
Brisbane: 2:00pm – 4:00pm
Darwin: 1:30pm – 3:30pm
Perth: 12:00(noon) – 2:00pm

Zoom link: https://bham-ac-uk.zoom.us/j/88405157711
Meeting ID: 884 0515 7711

Agenda

Start time	Item
**3:00pm (Sydney time)**	**Workshop opens**
3:00 – 3:20pm (20 min)	Welcome, introduction and housekeeping
3:20 – 4:00pm (40 min)	Group discussion 1: Stakeholder perspectives on priorities for regulation of products, services and information in the context of hearing health innovations
**4:00 – 4:10pm (10 min)**	**Break**
4:10 – 4:45pm (35 min)	Group discussion 2 (with vignettes to reflect on hypothetical scenarios)
4:45 – 5:00pm (15 min)	Summary and dissemination plan
**5:00pm**	**Meeting ends**

Advancing Regulatory Perspectives in Hearing Healthcare
Stakeholder Workshop

Topic guide for Group Discussion 1

* note that "people with hearing loss" has been chosen to encompass people who identify as Deaf, deaf, hard-of-hearing, hearing-impaired, or have just started to notice a loss of hearing acuity.

No.	Questions
1	**What upcoming changes or innovations** do you think are likely to have the **most impact** for people with hearing loss?
2	Can you list what you think would be the **main benefits** of these changes/ innovations for people with hearing loss?
3	Can you list **potential risks or drawbacks** of these innovations for people with hearing loss? For example, in terms of product safety, privacy, or accessibility of information or of care services?
4	What are your thoughts about **existing regulations** for hearing health information, products, and services in Australia?
5.	**What would be needed in terms of regulations** to ensure that upcoming hearing and communication information, services and products are safe, accessible, equitable and trustworthy, while preventing potential risks?

Group Discussion 2: Regulation and future trends in hearing healthcare

Vignette 1: Rony 39 years old

Consider the following scenario:

Aware that his hearing is not great, Rony has found information online and was able to order hearing aids at a fraction of the price that was advertised at the audiology clinic. He was also able to find the fitting software, so he can test his hearing and adjust the hearing aids himself. He can control the settings of the hearing aids via his phone, and it also gives him other useful information like his heart rate. Rony is not sure why he hears better with the hearing aids, but he does. He often has wax build-up and also has a tinnitus (ringing) on the right ear that has started just a year ago. He likes to be able to change the settings himself directly so he can cover the sound of the tinnitus.

TRENDS: THE INFORMED AND EMPOWERED CONSUMER & INCREASING PUBLIC AWARENESS AND DE-STIGMATIZATION
Consumers drive their own hearing care - Better informed, digitally savvy consumers, connected via social media, prefer to seek out assessment, treatment, and management options online over going to a hearing care provider.

A proliferation of in-ear devices changes popular conceptions of hearing aids. Instead of being just a prescribed medical product, devices are expected to improve people's hearing and measure additional health metrics with AI assistance. Because of the popularity of these devices, stigma towards hearing devices decreases.

(Based on: IDA Institute. Future Hearing Journeys Report)

Discussion:

When reading vignette 1 (Rony),

- what are the positives, drawbacks and risks that you can think of for Rony?
- what would be needed to keep the benefits in this scenario, and limit the risks?

**Vignette 2: Nellie 52 years old**
Consider the following scenario:<br><br>*Nellie has just been hired in a security firm and a company policy bans bringing mobile phones and any other recording devices into meeting rooms. She is unsure if she should mention that she uses hearing aids that connect directly to her phone and her smart home system. She is quite reliant on her hearing aids and she likes how her hearing aids respond and adapt to the type of noise in her environment. Her phone also has an integrated alarm detector in case she does not hear the fire or evacuation alarms. Her friends think that she should consider cochlear implants, but she has also heard of new drugs that could soon treat hearing loss. She prefers to wait until these drugs are available. She knows that cochlear implants would not bring normal hearing back anyway.*
TRENDS: DIGITAL TRANSFORMATION AND TECH INNOVATION<br>Hearing loss treated through medicine: Advances in science and medicine will create new treatments for hearing loss, including surgical, pharmaceutical, and gene therapy options.<br><br>Avatars and automation transform hearing care: Artificial intelligence becomes a tool used to diagnose uncomplicated cases of hearing loss. Person-centered care (PCC) is transformed and performed in new ways, assisted by avatars and virtual and augmented reality.<br><br>People with hearing loss will benefit from competitive prices and more options — hearing aids with AI assistance, over-the-counter devices, and non-medical tech. Decreasing regulation, however, leads to products of varying quality and opens questions about how data is handled.<br><br>*(Based on: IDA Institute. Future Hearing Journeys)*
**Discussion:**<br><br>When reading vignette 2 (Nellie),<br>- What are the positives, drawbacks and risks that you can think of for Nellie?<br>- What would be needed to keep the benefits and limit the risks?

**Vignette 3: Flick 25 years old**
Consider the following scenario:<br><br>*Flick has grown up deaf and has many deaf friends. She prefers using Auslan, but if that's not possible she can also manage orally, with her hearing aids. That takes much more effort and is tiring, but she can get by. Not everyone in her family or at work can sign. Because of under-developed auditory nerves, cochlear implant surgery would not give much more hearing than her hearing aids. She is ok with that. What she finds difficult however, is seeing all the ads about innovations that promise to "fix" hearing and those that warn about the impact of not hearing on cognition. She often feels judged and misunderstood for using more than one way to communicate and because she needs a range of adaptations, depending on the situations that she is in. Flick is not sure whether the hearing care provider she sees has recommended the best devices for her. Perhaps if she paid more for the hearing aids, she could get a better model?*<br><br>**TREND: INCREASED COMPETITION IN THE HEARING AID MARKET**<br>It is harder for independent health care professionals to turn a profit based on hearing aid sales.

They face competition from bigger chains buying up smaller chains and a consumer preference for wearable tech produced by electronics companies outside of the hearing aid market.

Consumer demands for transparency, trust, and unbundling of products and services - People with hearing loss adopt a consumer-driven mindset with expectations about convenience, simplicity, flexibility, sustainability, and quality that call for transparency. Consumers expect that they can trust their hearing care professionals (HCPs) to provide the best care and devices at the cheapest prices.

(Based on: IDA Institute. Future Hearing Journeys Report)

When reading vignette 2 (Flick),

- What are the positives, drawbacks and risks that you can think of for Flick?
- What would be needed to keep the benefits and limit the risks?

Workshop Ground Rules

To enable the workshop to run as smoothly as possible and to ensure all participants have a positive experience we kindly ask that you observe the following ground rules:

1. Consider that every guest in this workshop as a different expertise and background. We aim to learn from one another by putting the different pieces of expertise together.

2. We will not have enough time to do introductions, so we are asking all participants to introduce themselves in the chat. You do not have to provide your full name or your role if you do not wish. For example: “Hi I’m George, I’m the father of a deaf child and I volunteer for an advocacy group”, or “Hi I’m Lyn, I work for a hearing tech company and I am interested in this workshop because..”.

3. Enabling video is optional but we encourage you to leave your video on as a communication support for attendees who rely on or benefit from lipreading/speech reading.

4. Please keep your microphone muted unless it is your turn to speak.

5. If you would like to contribute to the discussion, please use the “raise hand” function and wait until invited to speak. You can then unmute yourself to speak.

6. The meeting will be recorded as stated in the Information Sheet.

7. To respect everyone’s privacy, and to meet the requirements of this project, you are not permitted to record or take photos during this workshop.

PARTICIPANT INFORMATION SHEET

ADVANCING REGULATORY SCIENCE IN HEARING CARE: STAKEHOLDER PERSPECTIVES (Version 2.0, 19-Aug-2022)

WHAT IS THE PURPOSE OF THIS STUDY?

Hearing care is undergoing a rapid transformation in line with innovations in digital healthcare, the development of advanced pharmacological therapies, and an increased focus on patient self-management. For example, the development of hearables (a subset of wearables) for hearing health and the approval of over-the-counter (OTC) hearing aids are helping to make technological intervention options for hearing loss more accessible.

Understanding the regulatory requirements for hearing innovation is key to ensuring hearing care is safe and effective and addresses the concerns of patients/consumers. However, given the system complexity and the pace of innovation, an understanding of key regulatory issues from multiple stakeholder perspectives, most importantly adults with hearing loss, is needed urgently.

WHY ARE WE APPROACHING YOU?

We are inviting individuals living in Australia and/or the United Kingdom (UK) who have expertise and/or an interest in the regulation of hearing care to take part in an online workshop. Your participation will help us to understand key issues facing the regulation of hearing innovations, including issues relating to patient benefit and burden, device classification (e.g., medical devices, hearables, personal sound amplification products (PSAPs), accessibility of care, privacy, artificial Intelligence (AI), sustainability, regulation for clinical trials and routine care and the marketplace.

WHAT WILL HAPPEN TO ME IF I TAKE PART?

If you take part in this research project you will be invited to attend a 2-hour online workshop. To help you prepare, you will be provided with written materials which will include the results of an online survey and a list of questions that will be discussed in advance of the workshop. It will take approximately 20 minutes to review the material ahead of the workshop. Communication support in the form of live captioning and BSL/Auslan interpreting will be available during the workshop. After the workshop, you will have no further involvement in the study.

WHO IS ORGANISING AND FUNDING THE RESEARCH?

This project is a collaboration between the Centre for Patient Reported Outcome Research, University of Birmingham and the Centre for Disability Research and Policy, The University of Sydney. Research funding for this study is provided by UK SPINE and the Centre for Regulatory Science and Innovation, Birmingham Health Partners.

WHO HAS REVIEWED THE STUDY?

The ethical aspects of this study have been approved by the University of Birmingham Research Ethics Committee [ERN_22-0712] and the Human Research Ethics Committee (HREC) of The University of Sydney [2022/491] according to the National Statement on Ethical Conduct in Human Research (2007).

DATA PROTECTION

In order to carry out the project described above, we will need to collect information about you, and some of this information will be your personal data. Under data protection law, we have to provide you with very specific information about what we do with your data and about your rights.

The University of Birmingham's web page 'Data Protection - How the University Uses Your Data' sets out much of this information, including how to ask any questions you may have about how your personal

data is used, exercise any of your rights or complain about the way your data is being handled. The rest of the key information you need to know about how we used your personal data is set out below.

Who is the Data Controller?
The University of Birmingham, Edgbaston, Birmingham B15 2TT is the data controller for the personal data that we process in relation to you.

What data are we processing and for what purpose will we use it?
All data collected from you during the consultations will be kept confidential by the researchers. Only researchers involved in the study will have access to the online survey data.

What is our legal basis for processing your data?
The legal justification we have under data protection law for processing your personal data is that it is necessary to do so for our project, which is a task we carry out in the public interest.

How long will my personal data be kept?
Your data will be retained by the University of Birmingham for 10 years after the publication of the project outcomes with the possibility to revisit and compare the de-identified data within that timeframe, depending on how the market and regulations in the field of hearing care evolve. After 10 years, the data will be securely destroyed. No data will be transferred outside of the University of Birmingham.

What will happen to the results of the project?
The results will be used to summarise key issues relating to innovations and regulatory guidance in the hearing sector, as perceived by stakeholders. We aim to publish the results in relevant scientific journals. You will not be identified in any report or publication. We can keep you informed on any publications arising from this study if you wish.

Can I change my mind about taking part?
Yes. Participation is voluntary. You can decide not to attend the workshop and you can also withdraw from the study during the workshop without giving a reason. However, your data will not be able to be removed or disintegrated after the workshop.

Can I withdraw my data?
No, you will not be able to withdraw your data.

Will I receive any compensation for participating?
If you are a person with hearing loss or a family member of a person with hearing loss, you will receive a £50 (or AUD $85) gift voucher for your participation in the workshop. If you choose to attend and subsequently withdraw, your compensation payment will be prorated for the time you participate in the workshop. Participation for other stakeholder groups is voluntary.

Who can I contact should I want to ask questions?
The project team will be happy to answer any questions you may have. Please see contact details below:

Primary point of contact:

In the UK:
Dr Sarah Hughes, Research Fellow, University of Birmingham
Email: s.e.hughes@bham.ac.uk
In Australia:
Dr Isabelle Boisvert, Senior Lecturer, The University of Sydney
Email: isabelle.boisvert@sydney.edu.au

Other contact details:
Principal Co-Investigator: Dr Sarah Hughes, Research Fellow, University of Birmingham
Email: s.e.hughes@bham.ac.uk
Principal Co-Investigator: Dr Isabelle Boisvert, Senior Lecturer, The University of Sydney
Email: isabelle.boisvert@sydney.edu.au

In the event of a complaint please contact:

In the UK:
University of Birmingham Research Ethics Officer: Susan Cottam
Email: s.l.cottam@bham.ac.uk | Phone: +44 (0)121 414 8825
In Australia:
The University of Sydney Human Ethics Manager
Email: human.ethics@sydney.edu.au | Phone: +61 2 8627 8176
